# Editorial: Movement Disorders and Sleep – Underlying Mechanisms, Clinical Aspects and Treatment

**DOI:** 10.3389/fneur.2019.01034

**Published:** 2019-10-17

**Authors:** Cristian Falup-Pecurariu, Nataliya Titova, K. Ray Chaudhuri

**Affiliations:** ^1^Department of Neurology, Faculty of Medicine, County Emergency Clinic Hospital, Transilvania University Braşov, Braşov, Romania; ^2^Department of Neurology, Neurosurgery and Medical Genetics, Pirogov Russian National Research Medical University, Moscow, Russia; ^3^Parkinson Foundation Centre of Excellence, King's College London, King's College Hospital, London, United Kingdom

**Keywords:** movement disorders, sleep, treatment, mechanisms, assessment

Nocturnal sleep dysfunction is a key problem in many movement disorders and one of the major non-motor symptoms in Parkinson's disease (PD) the world's fastest growing neurodegenerative disorder with consequences during waking hours at daytime as well as daily functioning (1). In PD for example, sleep dysfunction such as nocturnal waking and daytime somnolence was recognized by James Parkinson himself and continues to be a clinical challenge given the problems arise from the motor problems at night, neurotransmitter driven alterations in sleep architecture as well as drug related effects (2, 3). Non-motor endophenotypes of PD have been recognized and serotonergic dysfunction for instance in the raphe and limbic areas could drive aspects of sleep dysfunction in PD and some other related neurodegeneration (4).

In this special edition of the Frontiers in Neurology, sleep dysfunction in Movement Disorders is addressed focusing on possible pathophysiological mechanisms, clinical aspects of recognition, and awareness and treatment. Yousaf et al. provide a narrative review of the various types of molecular, structural, and functional neuroimaging techniques such as magnetic resonance imaging (MRI), functional magnetic resonance imaging (fMRI), diffusion tensor imaging (DTI), single-photon emission computed tomography (SPECT), and positron emission tomography (PET) that have been explored to provide evidence base for assessing structural, functional and neurochemical correlates of sleep disturbances in PD and other movement disorders such as Huntington's disease and Multiple system atrophy. These data might eventually lead to new drug discovery or repurposing of existing molecules to treat aspects of sleep dysfunction, currently a key unmet need.

Excessive daytime sleepiness (EDS) can be a disabling problem in PD, particularly in the more advanced stages although EDS is also recognized as a prodromal feature of PD and present all through the various clinical stages of PD (5, 6). In some the problem may even result in a clinical syndrome resembling narcolepsy without cataplexy with functional consequences such as sudden onset sleep episodes during driving which can be precipitated by some dopaminergic drugs (7). Biomarkers to detect this variant within PD is therefore, important and Ashraf-Ganjouei et al. describe a Diffusion MRI connectometry study in PD patients with EDS vs. those without and report decreased MRI based connectivity in the left and right fornix, left and right inferior longitudinal fasciculus (ILF), left inferior and middle cerebellar peduncles. Sherbaf et al. describe another diffusion MRI connectometry study in RBD as well as depression, a prodromal NMS, in 93 treatment-naïve and non-demented early PD and report that these two non-motor symptoms may be associated with lower connectivity in several white matter tracts with involvement of short association fibers (U-fibers).

Prodromal and pre prodromal stages of PD are emerging as key areas where early treatment initiation may be a priority and REM sleep behavior disorder (RBD) is a clinical biomarker for the development of α-synucleinopathies as well as a predictor of early cognitive decline (8). Excessive daytime sleepiness (EDS) can also be a prodromal non-motor symptom of PD. Gjerstad et al. address these two key sleep related symptoms in PD and discuss the implications in early stages of PD as well as the regulators of gene expression and core clock genes such as CLOCK and ARNTL (BMAL1) and the potential association of dopaminergic therapies and circadian genetic markers in PD.

Clinical assessment of sleep dysfunction is crucial for assessing the problem, addressing efficacy of treatment as well as providing validated outcomes for value based healthcare and in several papers, Rodríguez-Blázquez et al., Kurtis et al., and Skorvanek et al. discuss the roles, clinimetrics as well as application potential of clinical scales used to assess sleep dysfunction in PD and other movement disorders. Bhidayasiri et al. report an international study between Thailand, Japan, and India where they report on the utility of a PD Home Safety Questionnaire and discuss implications for adaptations based on a novel concept of concept of Personal (P)-Environmental (E) fit. Restless Legs Syndrome (RLS) often complicate sleep in PD and a link between the two conditions remain viable yet controversial. This issue is discussed by Ferrini-Strambi et al. who argue the case for well-designed systematic and strongly controlled longitudinal studies to clarify the aforesaid relationship.

Wearable sensors and the use of digital technology has become a topic of major research and clinical interest in the field of movement disorders. Sleep has been an increasing focus of research in this field and Madrid-Navarro et al. provide an interesting cross-sectional study using a wrist-worn device combined with machine-learning so as to process and detect circadian rhythms of sleep, motor, and autonomic disruption in PD providing an index to multidimensional circadian monitoring.

Advanced therapies in PD have now been shown to have beneficial or occasionally disruptive effects on sleep in PD. Sharma et al. review the role of deep brain stimulation at different anatomical targets which may affect the sleep-wake cycle via multiple factors, including motor symptoms, medication adjustment, and direct modulation of sleep-wake centers in PD.

This edition of the special Research Topic addressing sleep dysfunction in movement disorders therefore, present a series of cutting edge papers with original research data including neuroimaging and other biomarkers as well as reviews spanning clinical aspects of sleep dysfunction in PD and other movement disorders including RLS. We hope that the frontiers in sleep research in movement disorders would be facilitated by work presented in this special edition.

## Author Contributions

KR drafted and revised the manuscript for intellectual content. CF-P and NT revised the manuscript for intellectual content.

### Conflict of Interest

The authors declare that the research was conducted in the absence of any commercial or financial relationships that could be construed as a potential conflict of interest.
